# Harnessing Genome Editing Techniques to Engineer Disease Resistance in Plants

**DOI:** 10.3389/fpls.2019.00550

**Published:** 2019-05-07

**Authors:** Muntazir Mushtaq, Aafreen Sakina, Shabir Hussain Wani, Asif B. Shikari, Prateek Tripathi, Abbu Zaid, Aravind Galla, Mostafa Abdelrahman, Manmohan Sharma, Anil Kumar Singh, Romesh Kumar Salgotra

**Affiliations:** ^1^School of Biotechnology, Sher-e-Kashmir University of Agricultural Sciences and Technology of Jammu, Jammu, India; ^2^Division of Plant Biotechnology, Sher-e-Kashmir University of Agricultural Sciences and Technology of Kashmir, Srinagar, India; ^3^Mountain Research Center for Field Crops, Sher-e-Kashmir University of Agricultural Sciences and Technology of Kashmir, Srinagar, India; ^4^Department of Molecular Medicine, The Scripps Research Institute, La Jolla, CA, United States; ^5^Plant Physiology and Biochemistry Section, Department of Botany, Aligarh Muslim University, Aligarh, India; ^6^Department of Entomology, University of Arkansas, Fayetteville, AR, United States; ^7^Arid Land Research Center, Tottori University, Tottori, Japan; ^8^Botany Department, Faculty of Sciences, Aswan University, Aswan, Egypt

**Keywords:** CRISPR/Cas9, genome editing, homing endonucleases, phytopathogens, plant disease, stress

## Abstract

Modern genome editing (GE) techniques, which include clustered regularly interspaced short palindromic repeat (CRISPR)/CRISPR-associated protein 9 (CRISPR/Cas9) system, transcription activator-like effector nucleases (TALENs), zinc-finger nucleases (ZFNs) and LAGLIDADG homing endonucleases (meganucleases), have so far been used for engineering disease resistance in crops. The use of GE technologies has grown very rapidly in recent years with numerous examples of targeted mutagenesis in crop plants, including gene knockouts, knockdowns, modifications, and the repression and activation of target genes. CRISPR/Cas9 supersedes all other GE techniques including TALENs and ZFNs for editing genes owing to its unprecedented efficiency, relative simplicity and low risk of off-target effects. Broad-spectrum disease resistance has been engineered in crops by GE of either specific host-susceptibility genes (*S* gene approach), or cleaving DNA of phytopathogens (bacteria, virus or fungi) to inhibit their proliferation. This review focuses on different GE techniques that can potentially be used to boost molecular immunity and resistance against different phytopathogens in crops, ultimately leading to the development of promising disease-resistant crop varieties.

## Introduction

Plant-parasitic agents, such as pathogens and pests, are major yield-limiting factors causing 20–40% losses to global agricultural productivity thus posing significant challenges to food safety and security, which therefore remain a principal agricultural challenge worldwide (Savary et al., 2012; Rodriguez-Moreno et al., 2017). In the past decade, the introduction of the concept of genome editing (GE)/modification in crop plants revolutionized every aspect of plant science. Developing reliable and reproducible tools for GE in plants will have significant effects on basic as well as applied plant research. GE technologies accelerate functional analyses of genes and the introduction of novel traits into important crop plants. Site-specific endonuclease-based systems enable site-directed genome modifications by generating double-stranded DNA breaks (DSBs) in genes of interest with a very low risk of off-target (nonspecific cleavage) effects (Qi et al., 2013b; Khandagale and Nadaf, 2016). To date, four different site-specific endonuclease-based systems namely clustered regularly interspaced short palindromic repeats (CRISPR)/CRISPR-associated protein 9 (CRISPR/Cas9), zinc-finger nucleases (ZNFs), transcription activator-like effector nucleases (TALENs) and meganucleases (Osakabe et al., 2010; Baltes et al., 2015; Zaidi et al., 2016; Mushtaq et al., 2018) have been used extensively for crop improvement.

**FIGURE 1:**
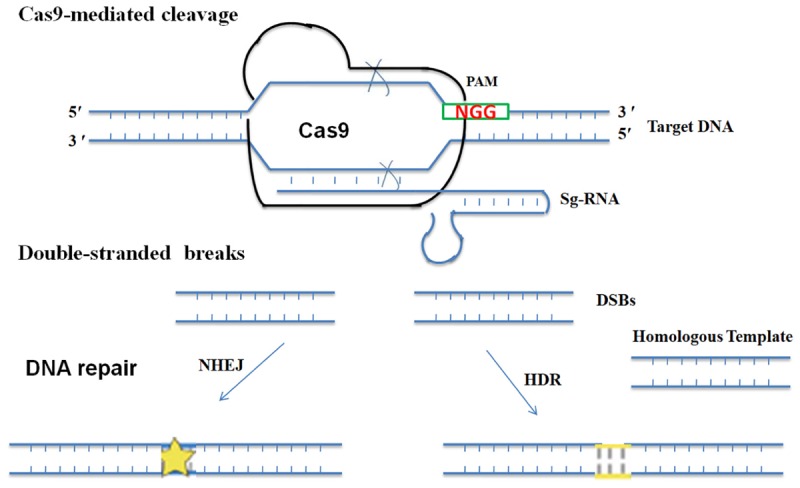
Brief overview of CRISPR/Cas9 system. Engineered CRISPR/Cas9 system depends on RNA guided nuclease, Cas9 to introduce double stranded breaks in target DNA. A single guide RNA, whose 20 nucleotides match the target DNA and a PAM (NGG or NAG, where N is any nucleotide) are essentially required for cleavage of the DNA in a sequence-dependent manner. Cas9 cleavage generates DSBs, which can be repaired through NHEJ or the HR pathway.

Modified nucleases are engineered to catalyze DSBs at a precise location in the genome, thus facilitating desired modifications of the DNA molecule at the target site (Sovova et al., 2016; Abdelrahman et al., 2018a). Subsequently, the cell’s own DNA repair machinery [homologous recombination (HR) and non-homologous end-joining (NHEJ) pathways] repairs the cut DNA. Genome break repair by NHEJ involves repairing the lesion by joining the two ends of DSB, which often leads to random indels of varying length and may cause frame shift mutation. HR requires exogenously supplied DNA sequences homologous to DSBs thus directing accurate repair of the DSB. Depending upon the nature of exogenously supplied DNA, a single nucleotide or larger genomic regions can be replaced via HR. Thus precise gene editing or targeted GE achieved via NHEJ or HR will potentially result in novel plant varieties with the addition of agronomically important traits or deletion of detrimental characteristics (Doudna and Charpentier, 2014; Abdelrahman et al., 2018a). Furthermore, as the recombination takes place in the cell itself with no foreign DNA involved in the repair mechanism, the variants become indistinguishable from the ones generated by classical breeding and this might serve as a lead to overcome the regulatory hurdles involved in the acceptance of transgenic crops (Pacher and Puchta, 2016). However, despite the fact that GE-induced mutations are indistinguishable from natural (e.g., solar radiation) or induced mutations (e.g., EMS, gamma irradiation), the European Court of Justice has declared products of this technology as GMOs^1^. It still remains a question whether genome edited crops without foreign DNA might escape the tough regulatory hurdles of GMOs and gain public acceptance or not. GE-technologies such as meganucleases, ZFNs, TALENs and CRISPR/Cas9 have revolutionized genome engineering. Unlike ZFNs, TALENs and meganucleases, CRISPR/Cas9 is independent of any protein engineering steps and can be retargeted to new DNA sequences by simply changing sequence of single-guide RNA. CRISPR/Cas9 requires a duplex-RNA structure [CRISPR RNA (crRNA): trans-activating crRNA (tracrRNA)] that guides Cas9 nucleases to the target DNA (Doudna and Charpentier, 2014; Khandagale and Nadaf, 2016; Ruiz de Galarreta and Lujambio, 2017). For efficient use, the dual crRNA: tracrRNA structure is engineered into a single-guide RNA (sgRNA) chimera targeted to specific genomic loci (Xing et al., 2014) (Figure 1).

**FIGURE 2:**
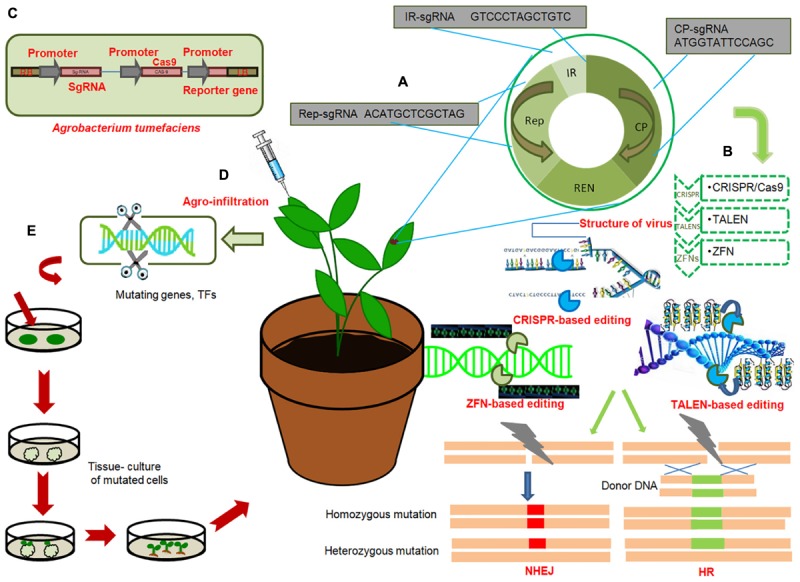
General work-flow of gene editing technologies to engineer disease resistance in crops **(A)** General genome organization of viruses; Target sgRNAs from each region of viral genome; replication associated protein (Rep), Intergenic region (IR), viral capsid protein (CP), with hypothetical sequences are shown in red. Multiplex genome editing strategy based on multiplex sgRNA targeting IR, CP and Rep of different viruses can be achieved by CRISPR/Cas9. **(B)** Illustration of three genome editing techniques conferring immunity of plants against virus: CRISPR/Cas9, TALENS, ZFNs. These technologies target different regions of viral genome and induce precise breaks at target sequences. Endogenous machinery of cells repair the breaks by non-homologous end joining (NHEJ) or homologous recombination (HR) thereby inducing genomic mutations at target locations. Induced mutagenesis in the viral or bacterial genome renders them ineffective. **(C)** T-DNA of *Agrobacterium tumefaciens* expressing sgRNA under CaMV-promoter, Cas9 protein under CaMV-promoter and reporter gene (GFP) under CaMV promoter. **(D)** Agroinfiltration of plant cells; injecting Agrobacterium containing engineered virus expressing sgRNA of target virus into Cas9-expressing plant. **(E)** Genome editing of genes or transcription factors, negatively regulating resistance against bacterial, viral or fungal pathogens, by deleting certain base pairs, in plants and subsequent raising of resistant plant by tissue culture techniques.

Different strategies have been adopted for boosting resistance against diseases in transgenic plants, such as detoxification of pathogen virulence factors, overexpressing resistance (*R*) genes and pathogenesis-related (PR) genes, increasing structural barriers and modification of defense-signaling pathways (Delteil et al., 2010; Wally and Punja, 2010; Du et al., 2018). Using modern omics platforms, susceptibility (*S*) or *R* genes were identified, providing many potential targets for improving crop protection (Barakate and Stephens, 2016; Singh et al., 2016; Yu et al., 2016; Ren et al., 2017). The unprecedented efficiency of GE techniques in editing the specific sequences of the *S* genes, which represent the best candidates for engineering resistance, has conferred disease resistance in various crops (Zhou et al., 2015; Jia et al., 2017; Das et al., 2019). Alternatively, genetic resistance in crop plants could also be enhanced based on multiplex CRISPR/Cas9 system, where a cassette of sgRNA is designed that can simultaneously edit or target most conserved regions of multiple viral genomes; and thus, interfering with their replication and movement (Iqbal et al., 2016) (Figure 2). In the present review, we evaluate the recent applications of various GE techniques to engineer disease resistance in plants and discuss how these tools could be used in the future to increase crop yields and improve quality.

## ZFNs: the First Developed GE Tool

Zinc-finger nucleases are synthetic restriction enzymes that can cleave any long stretch of double-stranded DNA sequences (Osakabe et al., 2010; Zhang et al., 2010; Carroll, 2011). ZFN monomer is an artificial nuclease engineered by fusing two domains: a non-specific DNA cleavage domain of the *Flavobacterium okeanokoites* I (*Fok*I) DNA restriction enzyme and a Cys_2_-His_2_ zinc finger domain (Curtin et al., 2011). Digestion of target DNA can be achieved when two ZFN monomers bind to their respective DNA target sequences. The two ZFN monomers will flank a 5- to 6-bp-long sequence within the DNA target sequence, allowing the *Fok*I dimer to digest within that spacer sequence. Upon dimerization, *Fok*I introduces a tailor-made DSB in the spacer sequence surrounded by two zinc finger array binding sites (Curtin et al., 2012; Puchta and Fauser, 2013). Endogenous DNA repairing machinery of the cell fixes this break by either stimulating error-prone NHEJ or the HR. If homologous sequences are absent, cell resorts to NHEJ, wherein broken ends are processed and joined directly, which often times may lead to either incorporation or deletion of nucleotides, causing a frameshift mutation in the gene and consequently its loss-of-function (Qi et al., 2013b).

Despite their complicated modular construction, ZFNs have already been used to tailor gene modifications in *Arabidopsis* (Osakabe et al., 2010; Petolino et al., 2010; Zhang et al., 2010; Even-Faitelson et al., 2011; Qi et al., 2013a), tobacco (*Nicotiana tabacum*) (Wright et al., 2005; Townsend et al., 2009), as well as crops, including maize (*Zea mays*), soybean (*Glycine max*) and canola (*Brassica napus*) (Shukla et al., 2009; Curtin et al., 2011; Gupta et al., 2012; Ainley et al., 2013). Resistance to bialaphos in maize (Shukla et al., 2009), resistance to herbicides in tobacco (Townsend et al., 2009) and ABA-insensitive phenotype in *Arabidopsis* (Osakabe et al., 2010) were achieved with ZFN technology. In the field of improving crop disease resistance, ZFNs have made little impact by editing host plant genes involved in disease development as they are complex to be engineered and difficult to be multiplexed (Khandagale and Nadaf, 2016; Ruiz de Galarreta and Lujambio, 2017; Jaganathan et al., 2018). Nevertheless, artificial zinc finger proteins (AZPs) have made a significant contribution to antiviral resistance in plants by blocking DNA binding sites of viral replication proteins (Sera, 2005; Takenaka et al., 2007). A report utilizing ZFN technology to boost disease resistance in crop plants was published by Chen et al. (2014), in which AZPs were designed to target a conserved sequence motif of begomoviruses. Multiple resistance against various begomoviruses, including *Tomato yellow leaf curl China virus* (TYLCCNV) and *Tobacco curly shoot virus* (TbCSV) was achieved by targeting a specific site in the viral DNA (Chen et al., 2014).

## Engineering Disease Resistance of Plants Based on the Talens

Transcription activator-like effector nucleases are transcription factors that are translocated by *Xanthomonas* bacteria through their type III secretion system into the plant cells (Boch and Bonas, 2010). TALEs can be engineered to bind any desirable DNA sequence that when fused to a nuclease (TALEN) can introduce DNA breaks at any specific location (Miller et al., 2011). The use of TALENs has been demonstrated at high efficiency in case of human cell lines and animals (Joung and Sander, 2013), but there have been only a few examples of TALEN applications in plants (Li et al., 2012; Sun et al., 2016). Moreover, most studies using TALENs to induce mutations through NHEJ which is often imprecise and can create mutations at targeted sites with loss-of-function (Joung and Sander, 2013). Rice bacterial blight is controlled by the interaction between TALE of *Xanthomonas oryzae* pv. *oryzae (Xoo)* and the host target *S* genes (Li et al., 2013). TALENs bind to the effector binding elements (EBEs) in the promoter region of the *S* genes, resulting in disruption of the EBEs and impairment of the molecular interaction between TALEs and the host *S* genes and subsequent improvement in disease resistance against *Xoo* strains (Li et al., 2012). Disease-resistant plants generated via TALEN-mutagenized *S* gene is similar to the mutant *S* gene *xa13* (recessive resistance allele of *Os8N3*) having mutation at the PthXo1 binding site in the promoter region of rice sucrose transporter encoding gene Os*SWEET11* (Yang et al., 2006; Streubel et al., 2013). Likewise, the *S* gene of rice *OsSWEET14/Os11N3* was mutated via TALEN, resulting in the production of disease-resistant rice with normal phenotypes (Li et al., 2012). Zhou et al. (2015) identified a sucrose transporter gene, namely *OsSWEET13*, a disease-susceptibility gene for the TALE effector PthXo2 of *Xoo2* strain, suggesting that the existence of cryptic recessive resistance to PthXo2-dependent *X. oryzae* pv. *oryzae* was resulted from a variation in the promoter regions of *OsSWEET13* in japonica rice. PthXo2-containing strains thus induce *OsSWEET13* in Indica rice IR24 due to the presence of an undescribed effector binding site which is not present in the alleles of Nipponbare and Kitaake cultivars of the *Oryza sativa* (japonica group).

*Xoo* injects TALEs that bind to EBEs in a sequence-specific manner, as a key virulence strategy to transcriptionally activate *OsSWEET14* gene (Zhang and Wang, 2013). Different TALEs, such as *AvrXa7, PthXo3, TalC*, and *Tal5* found in geographically distant *Xoo* strains, were targeted for TALEN-induced mutagenesis to compare the relative contribution of multiple EBEs within the SWEET14 promoter toward susceptibility to Xoo infection (Baufume et al., 2017). Baufume et al. (2017) reported the formation of an allele library of the *OsSWEET14* promoter regions via the expression of TALEN constructs in rice. They assessed the level of susceptibility in rice lines having AvrXa7-, TAL5- or TalC-EBEs in the promoter region of rice *OsSWEET14* gene. GE mutation using TALEN system, of the AvrXa7- or TAL5-EBE regions of rice *OsSWEET14* inhibited *Xoo* TALEs AvrXa7 and TAL5-mediated activation of SWEET14, resulting in disease resistance in the mutated lines (Blanvillain-Baufumé et al., 2017). The induction of sucrose transporter gene*- OsSWEET* in response to *BA13* wild-type bacteria which rely on *TalC* was prevented by indels within *TalC* EBE, in which the loss responsiveness of *TALC* failed to impart resistance to this strain. But, *TALC* EBE mutant line showed resistance to the strain which expresses an artificial SWEET14-inducing TALE whose CBE was also engineered in this line (Blanvillain-Baufumé et al., 2017). This study offered the first set of alleles engineered in *TalC* CBE and uncovered a broader activity for *TalC* as compared to *AvrXa7* or *Tal5*. Moreover, they proposed the presence of additional targets for *TalC* beyond SWEET14, which suggests that TALE-mediated induction of various, genetically redundant, host *S* genes may result in TALE-mediated plant susceptibility by a single effector.

Broad-spectrum resistance against begomoviruses has been evaluated by Cheng et al. (2015) using TALENs. TALENs were engineered using two conserved 12-nucleotide motifs into the *AC1* gene encoding a replication-associated protein (Rep) and inverted repeat genes of begomoviruses and were then tested with *TbCSV*, *TYLCCNV* and *Tomato leaf curl Yunnan virus* (*TLCYnV*). Results showed that the *Nicotiana benthamiana* which carries the TALEs conferred resistance to *TYLCCNV* and *TbCSV* but displayed partial resistance to *TLCYnV*. Recently, Shan et al. (2013) have shown that CRISPR/Cas9 can create efficient genome modifications in two model monocot plants rice (*O. sativa*) and *Brachypodium*. Further, the authors compared the frequency of mutation induced by CRISPR/Cas9 or TALENs, suggesting that CRISPR/Cas9 is more efficient to induce sequence-specific mutation in the respective plants (Shan et al., 2013).

## CRISPR/Cas9: a Pragmatic Approach Toward Development of Genome Edited Resistant Crops

**FIGURE 3:**
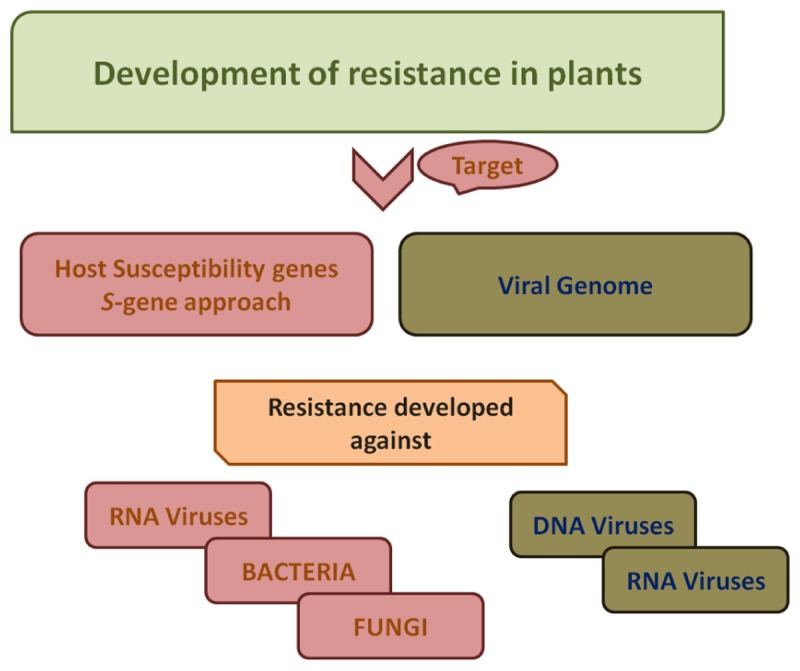
A schematic work-flow for the development of resistance in plants. For developing resistance against bacteria, fungi and RNA viruses, host susceptibility factors are targeted. Viral genome is targeted for development of resistance against viruses both DNA viruses as well as RNA viruses.

The increase in demand due to increasing population and environmental stress and concurrent challenge to meet it imposes an urgent need for novel strategies for improved crop production (Abdelrahman et al., 2018b). CRISPR/Cas9 system is a robust and versatile toolkit that uses sgRNA-engineered nucleases to make precise modifications at specified locations in the genome. Disease resistance in plants can be achieved by either editing genome of the pathogen or genes encoding susceptibility factors (*S*-genes) (Figure 3). To design sgRNA constructs, full length nucleotide sequences of viruses are retrieved from NCBI followed by selection of conserved sequences of viral genome bearing a 2–6 base pair DNA sequence immediately following DNA sequence targeted by the Cas9 nuclease in the CRISPR bacterial adaptive immune system known as protospacer adjacent motif (PAM) with minimum off-targets in plant genome (Iqbal et al., 2016).

## Developing Plant Resistance by Editing *S*-Genes–*S*-Gene Approach

Jiang et al. (2013) reported utilization of CRISPR/Cas9 as an effective strategy to induce mutagenesis in the promoter regions of bacterial blight *S* genes *OsSWEET14* and *OsSWEET11* in rice. Wang et al. (2014) use CRISPR/Cas9 in hexaploid wheat (*T. aestivum*) to introduce targeted mutations in the *TaMLO-A1* allele, one of three homoeoalleles that encode Mildew-Resistance Locus (MLO) proteins, resulting in improved disease resistance against downy mildew pathogen. Thomazella et al. (2016) used CRISPR/Cas9 to induce insertion/deletion mutation in tomato (*Solanum lycopersicum*) downy mildew resistance 6 (*SlDMR6-1*) gene. CRISPR/Cas9-induced mutation in *SlDMR6*-*1* enhanced disease resistance in tomato plants against different pathogens, including *Pseudomonas syringae pv. tomato, Phytophthora capsici*, and *Xanthomonas* spp. without any significant impairment in growth and development. Using the CRISPR/Cas9 technology, a non-transgenic tomato variety, Tomelo resistant to the powdery mildew fungal pathogen *Oidium neolycopersici* was generated by editing *slmlo1, a MILDEW RESISTANT LOCUS O* (*Mlo*) that confer susceptibility to this fungus (Nekrasov et al., 2017). Malnoy et al. (2016) targeted another such susceptibility gene, MLO-7, via direct delivery of purified CRISPR/Cas9 ribonucleoproteins (RNPs) to the protoplast of grape cultivar Chardonnay, to generate transgene free resistant powdery mildew grape cultivar.

Citrus canker caused by *Xanthomonas citri* subspecies *citri* is a serious disease for many citrus cultivars, leading to multitude of economic losses in various parts of the world (Jia et al., 2016). This bacterial pathogen injects its TALE_PthA4_, which binds to EBE motifs and transcriptionally activate the downstream target *canker susceptibility lateral organ boundaries 1* (*CsLOB1*) gene in the host, leading to disease-susceptibility (Jia et al., 2016). Out of its two alleles, promoter of Type I *CsLOB*1 was targeted for editing via epicotyl transformation of Duncan grapefruit and the mutation rate of 15.63% (#D13), 14.29% (#D17), 54.54% (#D18) and 81.25% (#D22), were obtained in transgenic plants (Jia et al., 2016). However, these transgenic lines showed canker symptoms similar to wild-type, suggesting that the activation of a single allele of *S* gene *CsLOB1*, either Type I *CsLOB1* or Type II *CsLOB1* by PthA4 is capable of causing disease of citrus canker and that mutations in the promoter region of both alleles of *CsLOB1* is required to generate plants having citrus canker resistance (Jia et al., 2016). Therefore, CRISPR/Cas9-induced mutation in both alleles of *CsLOBP* developed a citrus varieties-resistant to canker disease (Jia et al., 2016). In another study conducted by Peng et al. (2017) on Wanjincheng orange (*Citrus sinensis* Osbeck), CRISPR/Cas9-targeted modification of the *S* gene *CsLOB1* promoter was performed to improve the resistance of citrus toward citrus canker. The *CsLOB1* gene possesses two alleles in Duncan grapefruit and Wanjincheng orange. One allele harbors a G nucleotide (*CsLOB1^G^*) at the first site after the 3′ end of the PthA4 EBE in *CsLOB1* promoter while the second allele of the promoter lacks this nucleotide (*CsLOB1^-^*). Peng et al. (2017) demonstrated that CRISPR/Cas9-induced mutation of *CsLOB1^G^* alone was adequate to confer citrus canker resistance in Wanjincheng orange. These results are contradictory to the results of Jia et al. (2016) which suggested that inactivation of both alleles of *CsLOBP* is necessary for switching off *S* gene. However, Peng et al. (2017) in their report suggested that the anomaly may have risen as all citrus mutants obtained in the study of Jia et al. (2016) harbored only a 1-bp insertion which may not be sufficient to eliminate TAL-inducible expression of *CsLOB1.* Therefore, Peng et al. (2017) mutated more than 6 nucleotides in different transgenic plants, and deletion of the complete EBE _PthA4_ sequence from both *CsLOB1* alleles conferred a high degree of resistance to citrus canker. Moreover, the *CsLOB1* genes are heterozygous in citrus, the roles of *CsLOB1^G^* and *CsLOB1^-^* in different cultivars may be influenced differently which can be another possible reason (Peng et al., 2017). Jia et al. (2017) used CRISPR/Cas9 to edit *CsLOB1* in Duncan grapefruit. Both alleles of *CsLOB1* were targeted in the conserved region of the 1st exon, and rate of mutation of 31.58, 23.80, 89.36, 88.79, 46.91 and 51.12%, for six, *DLOB2, DLOB3, DLOB9, DLOB10, DLOB11*, and *DLOB12* lines, respectively was observed (Jia et al., 2017). The transgenic lines were resistant to canker with no off-target mutations and normal growth and developmental phases (Jia et al., 2017). Bastet et al. (2019) attempted to expand the resistance spectrum of the *Arabidopsis thaliana* eIF4E1 gene, a susceptibility factor to the Clover yellow vein virus (ClYVV), by mimicking the series of natural eIF4E alleles of *Pisum sativum*. The study showed resistance to ClYVV in A. thaliana requires only one or two mutations in eIF4E without impairing plant growth and development. Ortigosa et al. (2018) attempted to uncouple the SA-JA hormonal antagonism at the stomata for obtaining broad-spectrum resistance against *P. syringae* pv. *tomato* (*Pto*) DC3000, the causal agent of tomato bacterial speck disease without compromising resistance to the necrotrophic fungal pathogen *Botrytis cinerea*, causal agent of the tomato gray mold. *Pto* produces coronatine (COR) that stimulates stomata opening and facilitates bacterial leaf colonization in stomatal guard cells. *SlJAZ2*, a major co-receptor of COR was edited using CRISPR/Cas9 to generate dominant JAZ2 repressors lacking the C-terminal Jas domain, which prevent stomatal opening by COR.

## Developing Resistance Against Viruses by Editing Viral Genome

Chandrasekaran et al. (2016) developed broad viral resistance against Potyviruses (*Zucchini yellow mosaic virus* and *Papaya ringspot mosaic virus*) and Ipomovirus (*Cucumber vein yellowing virus*) in cucumber (*Cucumis sativus L.*) using CRISPR/Cas9 GE technology. Some plant RNA viruses like Potyviruses hijack *eukaryotic translation initiation factor 4E* (*eif4e*), a translational factor which has redundant functions in plants, to aid the execution of replication (Chandrasekaran et al., 2016). These viruses bind to *eIF4E* through virus-encoded protein (VPg), and mutations in this *eIF4E* gene impair the molecular interaction between the viruses and the host *S* gene causing quantitative variation in resistance phenotype (Chandrasekaran et al., 2016). Kis et al. (2019) utilized CRISPR/Cas9 system to inhibit an economically important, phloem-limited, insect-transmitted virus, *Wheat dwarf virus* (WDV), belonging to *Geminiviridae* family in Barley. Four sites within the genome of WDV were targeted and transgenic lines resistant to WDV were obtained. In a successive study, GE as a major tool for controlling the cotton leaf curl disease (CLCuD), which is caused by *begomoviruses* complex together with certain satellite molecules (α and β satellite), was proposed (Iqbal et al., 2016). Different techniques adopted to curtail proliferation of these begomoviruses were successfully circumvented by this complex genus, which include complex group of various viruses like *Cotton leaf curl Rajasthan virus* (CLCuRaV), *Cotton leaf curl Alabad Virus* (CLCuAlV), *Cotton leaf curl Kokhran virus* (CLCuKoV), *CLCuKoV-Bu* (Burewalastrain), *Cotton leaf curl Multan virus* (CLCuMuV), and *Cotton leaf curl Bangalore virus* (CLCuBaV) (Iqbal et al., 2016; Uniyal et al., 2019). GE technology using CRISPR/Cas9 system to induce mutation in the intergenic region (IR) and replication-associated protein (Rep) of the CLCuD-associated begomoviruses (CABs), can be adopted to confer wide-range of resistance against these viruses under natural conditions (Iqbal et al., 2016). Rice tungro disease (RTD), caused by the interaction between Rice tungro spherical virus (RTSV) and Rice tungro bacilliform virus is a serious constraint in rice production. Resistance to RTSV is a recessive trait governed by the translation initiation factor 4 gamma gene (eIF4G). Using CRISPR/Cas9 system mutations were induced in eIF4G of the RTSV-susceptible variety IR64, widely grown across tropical Asia (Macovei et al., 2018). This multifaceted genome engineering has added new horizons to unprecedented possibilities of resistance against intricate diseases where multiple pathogens complement each other in the development of diseases. An array of CRISPR edited crops to successfully circumvent different pathogens is listed in Table 1.

**Table 1:** Genome editing in plants to engineer resistance against phytopathogens.

S. No	Crop	Target Gene/Gene Mutated	Function of Gene	Resistance imparted to pathogen	Type of mutation	Transformation method	Effect on growth and development	Promotor used	References
**1.**	*Nicotiana benthamiana* and *Arabidopsis*	Candidate sites in the 3’ UTRs	Viral replication	*Cucumber mosaic virus* (CMV) or *Tobacco mosaic virus* (TMV)	InDel	Agroinfiltration in tobacco, Agrobac mediated transformation using floral dip in *Arabidopsis*	NA	35S- FnCas9 AtU6- SgRNA	Zhang et al., 2018
**2.**	Rice	Translation initiation factor 4 gamma gene *eIF4G*	Essential roles in the translation of viral RNA genomes	*Rice tungro spherical virus* (RTSV)	InDel	Agrobac mediated transformation of immature rice embryos	Insignificant	ZmUBI1-Cas9 AtU6- SgRNA	Macovei et al., 2018
**3.**	Tomato	*SlJAZ2*	Regulates stomatal opening	*Pseudomonas syringae* pv. *tomato* (*Pto*) DC3000	InDel	Agrobac mediated transformation of cotyledon segments	NA	ubiquitin promoter-Cas9, sgRNA	Ortigosa et al., 2018
**4.**	Tomato	*slmlo1*	Confers Susceptibility to powdery mildew	*Oidium neolycopersici*	InDel	Agrobac mediated transformation of cotyledon segments	Insignificant	35S- Cas9 AtU6- SgRNA	Nekrasov et al., 2017
**5.**	*Nicotiana benthamiana*	The helper component proteinase silencing suppressor (HC-Pro) and GFP sequences, Coat protein	Viral replication	*Turnip Mosaic Virus* (TuMV),	InDel	Agrobac mediated transformation and Agroinfection	NA	35S- Cas9 crRNAs - Pea early browning virus (PEBV)	Aman et al., 2018b
**6.**	Grapes	*MLO-7*	Confers Susceptibility to Powdery Mildew	*Erysiphe necator*	InDel	Delivery of purified CRISPR/Cas9 ribonucleoproteins (RNPs) to the protoplast	NA	NA	Malnoy et al., 2016
**7.**	Tomato	*DMR6* (2nd Exon) (downy mildew resistance 6)	Salicyclic acid homeostasis	*Pseudomonas syringae Pseudomonas capsici Xanthomonas sp.*	InDel Frame shift deletion	Agrobac mediated transformation of cotyledon segments	Insignificant	At U6-26 SgRNA 2^∗^35s Cas9	Thomazella et al., 2016
**8.**	Cotton^∗∗^	CABs, replication associated protein (Rep) and non-coding intergenic regions (IR), α-Satellite Rep and β-Sat IR.	Viral Replication	Cotton leaf curl disease (CLCuD) associated *Begomoviruses* (*CABs*) *and helper begomoviruses α and β satellites.*	InDel	Agroinfiltration or Agrobac mediated stable transformation	N.A	U6 (RNA Pol-3 promoter), CaMV 35S promoter for Cas9	Iqbal et al., 2016; Uniyal et al., 2019.
**9.**	Grapefruit Duncan (*Citrus paradisi Macf*.)	*CsLOB1* (Canker susceptible gene)	CsLOB1 is a member of the Lateral Organ Boundaries Domain (LBD) gene family of plant transcription factors.	*Xanthomonas citri subsp. citri* (*Xcc*)	Frame shift InDel	Agrobacterium mediated transformation of Duncan grapefruit epicotyls	Insignificant	CaMV 35S promoter for Cas9 and SgRNA, Nopaline synthase gene promoter for NptII, CsVMV, the cassava vein mosaic virus promoter for GFP.	Jia et al., 2017
**10.**	Rice (*Oryza sativa L.*)	*OsERF922* (Transcription factor)	Negative regulator of blast resistance in rice	*Magnaphorthe oryzae*	InDel	Agrobac mediated transformation of embryogenic calli	Insignificant	OsU6a rice U6a small nuclear RNA promoter (SgRNA), maize ubiquitin promoter (Ubi) (Cas9)	Wang et al., 2016
**11.**	Cucumber (*Cucumis sativus L*.)	*eIF4E* (eukaryotic translation initiation factor 4E) gene	Redundant Eukaryotic translational initiation factor essential for the Potyviridae life cycle.	Ipomovirus (*cucumber vein yellowing virus*), *Zucchini virus* (Potyvirus), *Papaya ring spot mosaic virus*	InDel	Agrobac mediated transformation of cucumber cotyledons	Insignificant	At U6(SgRNA) CaMV-35S (Cas9)	Chandrasekaran et al., 2016
**12.**	*Arabidopsis thaliana*	*eIF*(*iso*)*4E* (eukaryotic translation initiation factor 4E) gene	Recessive translational initiation factor usupered by potyviral protein VPg (viral protein genome-linked) to aid viral translation	*Turnip mosaic virus*	InDel	*Arabidopsis* plants dipped in the Agrobacterium suspension.	Insignificant	SgRNA (driven by PcUbi4-2 and AtU6-26 promoters, respectively), *Petroselinum crispum* (PcUbi4-2). used for Cas9	Pyott et al., 2016
**13.**	Citrus (Duncan grape fruit)	PthA4 effector binding elements (EBEs) in the Type-1 *CsLOB1* Promoter (EBEPthA4-*CsLOBP*) of the *CsLOB1* (*Citrus sinensis* Lateral Organ Boundaries) gene	Imparts Susceptibility to canker induced by the pathogenicity factor PthA4	*Xanthomonas Citri subsp. citri*	InDel	Agroinfiltration Agrobacterium mediated epicotyl transformation	N.A	CaMV 35S for both Cas9 and SgRNA.	Jia et al., 2016
**14.**	Wheat (*Triticum aestivum L.*,)	MLO (Mildew resistance locus)- A1 allele *TaMLO-A1* allele	Repress immunity against powdery mildew.	*Blumeria graminis f.sp. tritica* (*Bgt*)	InDel	Biolistic transformation of wheat embryos.	N.A	Maize Ub-1 promoter for Cas9, Wheat U6 promoter for SgRNA.	Wang et al., 2014
**15.**	*Nicotiana benthamiana*	Six target regions RBS (Rep binding site), hairpin nonanucleotide seq, three Rep motifs (1,2,3) essential for rolling circle replication	Essential for viral growth	*Bean yellow dwarf virus* (*BeYDV*)	InDel	Agroinfection	N.A	2^∗^35S(Cas9) AtU6 or At7SL RNA III promoter (SgRNA)	Baltes et al., 2015
**16.**	*Nicotiana benthamiana*	*TYLCV* Coding and Non-coding regions. Intergenic region (IR), viral capsid protein (CP), RCRII (rolling circle replication) motif of Rep.	Inplanta virus relication interference	*TYLCV* (*Tomato Yellow Leaf Curl Virus*), *Merremia mosaic virus* (*MeMV*), *beet curly top virus* (*BCTV*) *strain Worland*	InDel	Agroinfection of Tobacco rattle virus RNA2 genome having SgRNA into NB-Cas9OE plants	N.A	PEBV (Pea early browning virus) Promoter for SgRNA	Ali et al., 2015
**17.**	*Arabidopsis* and *Nicotiana benthamiana*	At7, B7 and C3 sites and several sites of Viral genome	Essential for Viral replication	*Beet Severe Curly Top Virus* (*BSCTV*)	InDel	Agroinoculation, Agrobac mediated transformation	N.A	2^∗^35S (Cas9) At U6 (SgRNA)	Ji et al., 2015
**18.**	*Nicotiana benthamiana*	coat protein (CP), RCRII domain of replication associated protein (Rep).	Essential for viral growth and replication.	*Cotton Leaf Curl Kokhran Virus* (CLCuKoV) and *Merremia mosaic virus* (*MeMV*)	InDel	Agroinfection of Tobacco rattle virus RNA2 genome having SgRNA into NB-Cas9OE plants	N.A	PEBV (Pea early browning virus) Promoter for SgRNA	Ali et al., 2016
**19.**	Barley	Rep, MP, LIR	Essential for viral growth and replication	*Wheat dwarf virus* (WDV)	InDel	Agrobacterium mediated	Insignificant	Cas9-Ub1	Kis et al., 2019
**20.**	*Arabidopsis*	eIF4E1 gene	Susceptibility factor	*Clover yellow vein virus* (*ClYVV*).	single base substitution	Agrobacterium mediated	Insignificant	nCas9At-PmCDA1At- AtU6	Bastet et al., 2019
**21.**	Banana	Integrated genome of banana streak virus (eBSV) in the banana genome	Viral growth and development	*Banana streak virus* (*eBSV*)	InDel	Agrobacterium mediated	Insignificant	PcUbi-Cas9 OsU6 P- sgRNA	Tripathi et al., 2019

*^∗∗^Concept of CRISPR/Cas9 as a tool to Circumscribe Cotton Leaf Curl Disease has been conceived by Iqbal et al. (2016) and Uniyal et al. (2019). However, the experimental evidences are yet to be published.*

Ji et al. (2015) were successful in inhibiting the accumulation of *beet severe curly top virus* (BSCTV) up to 70% in *A. thaliana* and *N. benthamiana* by efficiently targeting specific regions in the viral genome using CRISPR/Cas9 system. Likewise, Ali et al. (2016) utilized CRISPR/Cas9 strategy to induce mutation in the non-coding IR of three geminiviruses: *Merremia mosaic virus* (MeMV), *Tomato yellow leaf curl virus* (TYLCV) and *beet curly top virus* (BCTV) and that were subsequently degraded and rendered unfit for replication. Baltes et al. (2015) utilized six regions within the genome of *bean yellow dwarf virus* (BeYDV). The regions are Rep binding site (RBS), hairpin, nonanucleotide sequence and three Rep motifs considered essential for rolling circle replication (motifs I, II and III), for restricting *Bean yellow leaf curl virus* infection with the CRISPR/Cas system (Baltes et al., 2015). CRISPR/Cas 9 gene editing has also been used to inactivate an integrated endogenous banana streak virus (eBSV) in the B genome of plantain (AAB), an economically important sub-group of banana. Stress and *in vitro* culture activates latent eBSV in the B genome and mutation in the targeted sites prevent proper transcription and translation into functional viral proteins (Tripathi et al., 2019). Zhang et al. (2018) reprogrammed and expressed the CRISPR-Cas9 system from *Francisella novicida* (FnCas9) and its RNA-targeting guide RNA in *N. benthamiana* and Arabidopsis plants to confer resistance to two positive-sense RNA plant viruses, cucumber mosaic virus (CMV) and tobacco mosaic virus (TMV). FnCas9 edited plants exhibited significantly attenuated virus infection symptoms and reduced viral RNA accumulation.

Clustered regularly interspaced short palindromic repeat (CRISPR)/CRISPR-associated protein 9 use has moved forward from engineering disease resistance in host plants to dissection of the function of genes in the pathogen involved in plant infection. An oomycete pathogen of soybean *Phytophthora sojae* was considered as a model organism for studying the genetics of oomycete pathology and physiology owing to its economic importance. An effector gene of *P. sojae*, RXLR effector gene *Avr4/6* identified by soybean R genes *Rps6* and *Rps4* was edited using CRISPR/Cas9 system to find its possible role in pathogenicity (Fang and Tyler, 2016; Shanmugam et al., 2019). This study provided valuable insight toward functional analysis of this plant pathogen opening new avenues for understanding the pathogenomics of other economically important pathogens as well. GE using CRISPR/Cas9 also confirmed that the point mutations G770V and G839W, as well as a novel mutation, 5N837, in oxysterol binding protein related protein-1 (ORP1) gene confer resistance to oxathiapiprolin in *P. capsici* and *P*. *sojae* (Miao et al., 2018). Zhao et al. (2018) utilized CRISPR/Cas9 to function validate *Ptr* gene, a constitutively expressing atypical resistance gene which confers broad spectrum blast resistance. A two base pair (bp) deletion within the *Ptr* protein coding region rendered the mutant line M2354 susceptible to *M. oryzae* suggesting its role in blast resistance. A stable *F. oxysporum*-optimized CRISPR/Cas9 system has been developed using a *F. oxysporum*-optimized Cas9/sgRNA ribonucleoprotein (RNP) and protoplast transformation method. This system has efficiently disrupted two genes, *URA5* and *URA3* generating uracil auxotroph mutants that are resistant to 5-fluoroorotic acid, 5-FOA. In addition, a polyketide synthase gene *FoBIK1* was also disrupted which confirmed the role of the gene in the synthesis of the red pigment, bikaverin (Wang et al., 2018).

## Engineering Broad-Spectrum Disease Resistance in Crops With GE Technologies

Genome editing techniques hold tremendous potential for improvement of disease resistance in the crops. Systematic analysis of the genes followed by engineering of the pathways that will boost molecular immunity in plants can be achieved by GE techniques. With the impressive progress in next-generation sequencing (NGS) and evolution of various NGS platforms as molecular microscope beside parallelization of the sequencing reaction has deeply augmented the total number of sequences produced of various pathogens and plants (Buermans and den Dunnen, 2014). A plethora of techniques are available to elucidate the genes and pathways involved in pathogen resistance. Transcriptomic analysis of crop and pathogen provides valuable insight into defense and virulence pathways of both crop and pathogen. A repertoire of information generated by studying expressional changes in proteins, protein modifications, protein–protein interactions during the plant–pathogen interaction can be used to unravel key proteins involved in the pathogenesis. With this wealth of information, modification or alteration the genome of crop or pathogen, resulting in disease suppression can be achieved (Barakate and Stephens, 2016).

Viral diseases are difficult to control and application of chemicals does not eradicate the diseases. GE techniques can be beneficially utilized to develop strategies of viral interference in most of the devastating and economically important viral diseases in crops. Ilardi and Tavazza (2015) have proposed GE techniques as effective tools for controlling a *Plum pox virus* (PPV), a devastating pathogen that affects stone fruits. PPV primarily affects fruits, resulting in change in color, texture, weight, malformation and reduction in their nutritive value. Several strategies based on the usage of qualified PPV-free plant material, regular orchards surveys, eradication of diseased trees and treatment with insecticides to manage aphid populations had achieved little success in controlling PPV (Ilardi and Tavazza, 2015). GE techniques can target specific positions in the genome of PPV that are essential for its growth and replication, thus rendering it unfit to attack stone fruits thereby successfully rescuing stone fruit industry.

Fungal and bacterial diseases are curtailing production of crop plants worldwide. Qualitative or quantitative resistance can be engineered to a specific race of the pathogen by exploiting the fact that race-specific resistance can be achieved through the deliberate introduction of R-genes. In addition, antimicrobial genes, pathogen virulence detoxification, PR genes, increase in barriers of structural nature, RNAi and the defense-signaling pathways modification can significantly contribute to control of the plant diseases (Wally and Punja, 2010). A comprehensive review on designing and engineering novel synthetic R-genes combining several pathogen recognition sites (PRSs) which enable plant to increase resistance against conserved pathogen effectors and/or PAMPs through GETs has been published by Andolfo et al. (2016). Mutation in the active sites of both nuclease domains, RuvC and HNH of Cas9, known as dead endonuclease (dCas9) render it inactive. However, dCas9 still retains the aptitude of DNA binding at sites defined by the guide RNA sequence and the PAM. This catalytically inactive Cas9 can be exploited to repurpose targeted gene regulation on a genome-wide scale. dcas9 can be fused to regulatory elements viz proteins or RNA molecules for blocking elongation of transcription, binding of RNA polymerase, or transcription factor. This technique known as CRISPR interference (CRISPRi) can control activation or down-regulation of transcription which depends on the specific site(s) recognized by the complex dCas9–guide RNA. dcas9 can also be used to recruit any major DNA modification domains or chromatin-remodeling complexes, which includes histone acetylases and deacetylases, methylases and demethylases, DNA methylases and demethylases, Swi-Snf, kinases and phosphatases, and others to enable targeted epigenetic changes to genomic DNA (Doudna and Charpentier, 2014). CRISPRi can also be exploited to repurpose changes in the genome of necrotrophs to successfully establish immunity against toxins and cell-wall-degrading enzymes (Wally and Punja, 2010). Genes encoding these harmful factors can be modified at a genomic DNA level as opposed to gene knockdown at mRNA level, as in case of host-induced gene silencing (HIGS), therefore may reduce the chances of incomplete knockdowns or unpredictable off-targeting comparatively. However, detailed studies should be carried out to compare the efficiency of the two techniques. Wally and Punja (2010) listed some genes that can be potential targets for boosting immunity in plants. Several virulence genes such as the oxalic acid encoding genein *Sclerotinia sclerotiorum*, Trichothecene mycotoxin like *deoxylnivalenol* (*DON*) encoding gene in *Fusarium culmorum* and *Fusarium graminearum*, the causal agents of *Fusarium head blight* (*FHB*), a devastating disease of cereals, can be targeted (Wally and Punja, 2010). Immune receptors can also be engineered by Gene editing Techniques (GETs) to increase the spectrum of recognition specificities of pathogens by crops which in turn can substantially contribute to crop improvement. A detailed description of transferring and engineering of immune receptors in crops to improve recognition capacities has been reviewed by Rodriguez-Moreno et al. (2017).

## Recent Developments in CRISPR Based Gene Editing

Early findings of GE via CRISPR/Cas9 in mammalian cells established relatively high off-target potential (Hsu et al., 2013; Tycko et al., 2016). Off-targeting may hamper potential applications of CRISPR, especially in case of gene therapy. Various approaches have been developed to identify off-target effects in human cells, such as Digenome-seq, GUIDE-seq, HTGTS and BLESS (Yin et al., 2017) and these tools can be utilized in plants for better evaluation of Cas9 specificity on a genome-wide scale. In case of plants sequencing of potential off-target sites identified by using various bioinformatic approaches showed no off-target cleavage (Nekrasov et al., 2017). Similarly, in a study conducted by Feng et al. (2014), CRISPR/Cas9-induced mutant subjected to whole genome sequencing could not detect any off-target effects, thus showed high specificity in plants. However, off-target cleavage has been reported in rice, maize and soyabean mainly occurring in gene paralogs with almost identical sequences to the targets (Li et al., 2016). CRISPR-Cas9 specificity has been evaluated by biased off-target detection (Zhang D. et al., 2016). In a study, 13 putative off-target sites for three sgRNAs in rice were sequenced in which only one off-target was detected that harbored a single mismatch distal to the PAM (Zhang F. et al., 2014; Zhang H. et al., 2014). Another study conducted in wheat also revealed that the off-target frequency of CRISPR-Cas9 was very low (Zhang Y. et al., 2016). Recently, off-targeting of CRISPR-Cas9 was shown to be reduced in a study using preassembled complexes of purified Cas9 protein and guide RNA (ribonucleoproteins complexes or RNPs) into lettuce protoplasts (Woo et al., 2015). Interestingly, deep sequencing barely detected the off-target effects, when RNPs were used for editing, supporting the view that this approach enhances the specificity of CRISPR-Cas9 in plants (Svitashev et al., 2015; Woo et al., 2015; Liang et al., 2017). A great advantage of the use of RNPs is that it reduces the chimera or mosaic modifications in progeny plants. In order to detect potential off-targets and to design sgRNA in plants, different bioinformatic tools, such as CasOT (Xiao et al., 2014), CRISPRMultiTargeter (Prykhozhij et al., 2015) and Cas-OFFinder (Bae et al., 2014) are used.

Off-target effects based on GE may not cause serious issues for plant breeding as compared to physical and chemical mutagenesis used in conventional breeding program that produce many mutations in each plant (Salvi et al., 2014). Off-targeting is commonly low in plants as compared to other organisms and the undesired mutations can be eliminated by backcrossing (Bortesi et al., 2016). Nevertheless backcrossing is time consuming that would slow down the progress in crop improvement. Furthermore, off-targeting of CRISPR-Cas9 system could raise regulatory concerns in genome-edited plants.

**FIGURE 4:**
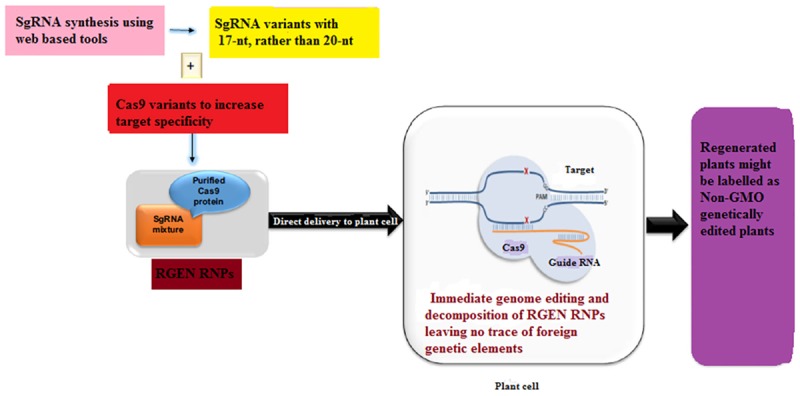
A proposed scheme for the development of a transgene-free food crop using genome editing. Non-homologous end-joining (NHEJ)-mediated plant breeding proceeds in the following manner. After designing the targeting domain of ZFNs and TALENs (guide RNAs of the CRISPR/Cas9 system), the specificity and off-target effect are validated in plant cell cultures. Plant cells modified by highly specific ZFNs, TALENs and CRISPR/Cas9 are subjected to an initial screen focused on on-target gene modifications. In addition to the acquired trait, the potential environmental impacts of the plants are evaluated in a laboratory. If the plants have an implication in environmental risks, such as the emergence of herbicide-resistant weeds by hybridization, test cultivation is carried out in an isolated field to evaluate their risks to the environment carefully. Moreover, the food product derived from such crops is subject to food safety assessment. If the plants have no implication in environmental risks, such plants are cultivated in a common field.

Plants transformed with CRISPR-Cas9 system may contain redundant insertions of plasmid DNA at both desirable and undesirable sites in the genome while harboring insertions and/or deletions at the target site (Kim et al., 2014). These plants may limit the use of GE in plant sciences and sustainable agriculture as these plants are often considered to be genetically modified organisms (GMOs) and may be subjected to tough GM laws in some countries. Even though the foreign DNA can be removed by genetic segregation but this is not feasible in asexually reproducing plants. Since recombinant DNA constructs have been used in the production of gene-edited plants, some local regulatory authorities do not accept even the edited plants from which foreign DNA has been removed (Sprink et al., 2016). Until now two DNA-free GE approaches have been described in case of plants which involve the delivery of a mixture of Cas9-encoding mRNA and gRNA (Zhang D. et al., 2016) or pre-assembled ribonucleoproteins (RNPs) (Figure 4) (Yin et al., 2017). On the other hand, the efficiency of transient expression of CRISPR-Cas9 RNA is relatively low, signifying that further optimization is needed. In this case, one promising approach would be to add some protectant to stabilize the RNA (Latorre et al., 2016). In an non-transgenic approach, Sauer et al. (2016) efficiently generated transgene-free protoplasts via CRISPR GE in each of the two enolpyruvylshikimate-3-phosphate synthase (EPSPS) genes in flax.

Nonetheless another non-transgenic approach used engineered RNA virus-based expression vectors to deliver Cas9 and sgRNAs in order to produce plants free of foreign DNA. In view of the fact that viral DNA does not integrate into the plant genome, thus the foreign DNA is not transmitted through the germline to the next generation (Ali et al., 2015). This approach was used to deliver sgRNA targeted to PDS gene with *Tobacco rattle tobravirus* (TRV)-based vector in N. benthamiana (Fondong, 2017). Certainly, further improvements in non-transgenic CRISPR induced gene editing will hasten the field of plant genome engineering, not only to combat viruses, but also to tackle other challenges as well.

Clustered regularly interspaced short palindromic repeat/CRISPR-associated protein 9 system definitely possesses exceptional potential for plant GE. However, its efficiency and specificity can be limited by some factors such as PAM specificity, design of sgRNA and off-target activity (Langner et al., 2018). Therefore, a number of strategies have been developed to improve Cas9 specificity including use of truncated sgRNAs (Scheben et al., 2017), recently identified highly reliable Cas9 variants (Slaymaker et al., 2016) and paired nickases (Barakate and Stephens, 2016). Several key factors limit the Cas9 specificity such as the nature of PAM sequence located immediately downstream of the protospacer element and the tolerance of mismatches in the PAM distal region. The range of target sequences is limited by stringent of the Cas9PAM sequence (NGG sequence). Although, Cas9 tolerates the multiple mismatches in the PAM distal region that significantly reduce its affinity to the target site (Fu et al., 2013). Since NGG-PAM sequences are frequently found in plant genomes, there may be complexity in targeting specific genomic regions with these sequence constraints, particularly in case of highly AT-rich genomes (Zetsche et al., 2015). The discovery of alternative PAM sequences generated by introducing mutations into the PAM-interacting domains of wild type SpCas9 overcame the target range limitations by the NGG-PAMs (Kleinstiver et al., 2015). This PAM sequence includes motifs such as NGAG (Cas9 variant VQR, D1135V/R1335Q/T1337R), NGAG (Cas9 variant EQR, D1135E/R1335Q/T1337R) and NGCG (Cas9 variant VRER, D1135V/G1218R/R1335E/T1337R) (Anders et al., 2016). Cas9 specificity and the number of genomic loci that are amenable to targeting by CRISPR/Cas9 can be increased by newly discovered enhanced protospacer region in NmCas9 (24 nts instead of 20 bp) (Hou et al., 2013). Furthermore, the use of CRISPR endonuclease cpf1 (also known as Cas12a) recently identified and characterized from *Prevotella* and *Francisella* (Zetsche et al., 2015) overcame some of the shortcomings of Cas9 enzyme such as its G-rich PAM requirement. Thus cpf1 can greatly expand the number of target sites available for GE as it has the ability to recognize T-rich PAM site. This enzyme is not only useful for targeting AT-rich genomes, but it can also be applied to phenotype or disease-linked mutations in AT-rich regions through homology-directed repair. Moreover, it creates a staggered double stranded DNA cut with a 5′ overhang and does not require tracrRNA for function. Hence, CRISPR-Cpf1 can be used to introduce virus-resistance amiRNAs, tasiRNAs or other RNA silencing cassettes at the precise genomic loci in order to improve the overall expression and performance of transgene because of the relative ease of inserting genes. Since CRISPR-Cpf1 GE platform is advantageous over CRISPR-Cas9 system, it can be a formidable tool in crop improvement and handy in generating durable plant virus resistance. Most recently, an efficient and much more precise RNA targeting and editing tool, CRISPR-Cas13a (previously known as C2c2) was characterized in prokaryotes that can be recruited in plants to confer resistance against RNA viruses and regulate gene expression (Khan et al., 2018). Aman et al. (2018a) used CRISPR/LshCas13a to confer resistance against plant RNA virus, thus demonstrating that this system can be applicable for future crop improvement. Such kind of novel CRISPR-Cas systems offer unprecedented genome-editing tools for combating plant viruses.

Clustered regularly interspaced short palindromic repeat-based systems usually produce double stranded breaks (DSBs) resulting in mutants with either gene replacements or insertions [via homology-directed repair (HDR)] or gene knockouts (via NHEJ) (Zetsche et al., 2015). Base editing is a distinctive GE system that involves site-specific modification of DNA without requiring DSBs, or donor templates, or depending on HDR and NHEJ (Hess et al., 2017). The base editors such as BE3 (Komor et al., 2016), BE4 (Komor et al., 2017), targeted AID (Nishida et al., 2016), and dCpf1-BE (Li et al., 2018) have already been used in various cell lines and organisms (Yang et al., 2017). These base editing systems use Cas9 or Cpf1 variants to recruit cytidine deaminases that generate C to T substitutions by exploiting DNA mismatch repair pathways. In a study, herbicidal gene, *C287* in rice was base-edited using activation-induced cytidine deaminase (targeted AID method) in which dCas9 fused with cytidine deaminase was used for base editing without requiring DSBs or donor templates (Li et al., 2018). More recently, the third generation of editors, BE3 was used for base editing of rice *OsPDS* and OsSBE genes and demonstrated the successful application of base editing in rice (Jaganathan et al., 2018). BE3 uses a Cas9 nickase (Cas9n) (makes single-strand cuts/nicks in DNA), cytosine deaminase and uracil glycosylase inhibitor (inhibits base-excision repair) and the edit is propagated by controlling cell’s repair mechanism. Fourth generation base editors (BE4) involve modifications such as an additional copy of a repair inhibitor and are more efficient than BE3 and target-AID (Marx, 2018). Plant biologists have expanded their base-editing toolbox beyond the conversion of cytosine to thymine for point-mutagenesis experiments. Recent development of adenine base editors (ABE) by fusion of an evolved tRNA adenosine deaminase (Escherichia coli TadA) with SpCas9 nickase (D10A) were shown to generate A-T to G-C conversions when directed bysgRNAs to genomic targets in human cells (Gaudelli et al., 2017). Researchers at various Korean research institutes adapted ABEs to mediate the conversion of A-T to G-C in protoplasts of *A. thaliana* and *B. napus* and demonstrated the successful application of ABE system to protoplasts through transient transfection and to individual plants through Agrobacterium mediated transformation to obtain organisms with desired phenotypes (Kang et al., 2018). Recently, base editing has been demonstrated to be highly efficient system in creating targeted point mutations at multiple endogenous loci in rice and wheat (Li et al., 2018). Additionally, base editing can be used to develop virus resistance in plants by targeting virus genome and generate stop codons through CRISPR-stop (Kuscu et al., 2017) or iSTOP (Billon et al., 2017) resulting in generation of nonfunctional proteins and thus restricting the pathogen propagation and systemic spread across plant tissues. Similarly, base editing can be employed to develop plants with immunity against different single and multiple pathogens by targeting and modifying the genome. Thus, base editing can open up new avenues for plant genome engineering and biotechnology. Moreover, multiplex genome engineering of a potentially unlimited number of genes is now possible by CRISPR/Cas9. Furthermore, different CRISPR systems can be combined to enhance the capacity of multiplex genome engineering (Zhang H.Y. et al., 2017).

## Future Considerations

The toolbox of GE techniques provides a ground for designing strategies to overcome the devastating phytopathogens. These GE techniques can play a vital role in providing molecular immunity against the broad-spectrum of phytopathogens, by altering the genes that confer susceptibility toward the pathogen. Specific regions of the viral genome involved in the viral replication can be targeted to curb the menace of devastating plant viruses. Such systems can also be multiplexed to target multiple DNA viruses. Bacterial and fungal disease resistance can also be engineered in different crops, thereby improving crop productivity. It can also offer insight into the molecular mechanism of pathogenesis of a virus or bacteria, by specifically knocking down or knocking out different genes involved in pathogenesis. Insight into key players establishing plant–pathogen interactions, the involvement of different signaling molecules, receptor proteins can also be elucidated by GE of potential target genes. Epigenome of plants, as well as phytopathogens, can also be targeted to boost genetic resilience in crops. Off-target effects and random integration of DNA insert from plasmid vectors into host genome limit its wider applicability (Koo et al., 2015). Moreover, stable integration of plasmid-mediated RGENs in the host genome might result in detrimental effects and cytotoxity (Woo et al., 2015). Advancements in GETs such as developing approaches to directly transfer Cas9 RNPs and/or donor DNA into protoplasts for gene editing can efficiently be used to generate mutations in genes of interest without leaving vector sequences in the genome. However, robust evaluation of promising edited plants in the field for several generations must be carried out to ensure that mutations are stable and to rule out the negative impact of gene editing on plant growth and development. It is necessary to spread the message of its applicability and implementation across farmers and the commodity groups to generate awareness toward any ethical concerns they may have.

## Author Contributions

All authors listed have made a substantial, direct and intellectual contribution to the work, and approved it for publication.

## Conflict of Interest Statement

The authors declare that the research was conducted in the absence of any commercial or financial relationships that could be construed as a potential conflict of interest.

## Footnotes

1 https://www.nature.com/articles/d41586-018-05814-6
